# A phase II study of active specific immunotherapy and5-FU/Leucovorin as adjuvant therapy for stage III colon carcinoma

**DOI:** 10.1038/sj.bjc.6600254

**Published:** 2002-04-22

**Authors:** A Baars, A M E Claessen, J Wagstaff, G Giaccone, R J Scheper, S Meijer, M J A G Schakel, H E Gall, C J L M Meijer, J B Vermorken, H M Pinedo, A J M van den Eertwegh

**Affiliations:** Department of Medical Oncology, Vrije Universiteit Medical Center, Amsterdam, The Netherlands; Department of Pathology, Vrije Universiteit Medical Center, Amsterdam, The Netherlands; Department of Surgery, Vrije Universiteit Medical Center, Amsterdam, The Netherlands

**Keywords:** active specific immunotherapy (ASI), colon cancer, chemo-immunotherapy, delayed type hypersensitivity (DTH), autologous tumour cell vaccines

## Abstract

Active specific immunotherapy, using vaccines with autologous tumour cells and BCG, significantly reduces the rate of tumour recurrence in stage II colon cancer patients, while no clinical benefit has yet been observed in stage III patients. Adjuvant treatment with 5-Fluorouracil/Leucovorin is now considered standard therapy for stage III colon carcinoma and results in an absolute survival benefit of approximately 10%. Yet, the 5-year overall survival rate of stage III colon cancer patients is only 40–50%. Combining chemotherapy and immunotherapy might improve prognosis for stage III patients, especially when considering that active specific immunotherapy and chemotherapy have shown synergistic effects in pre-clinical tumour models. We performed a phase II study with 56 patients, using the combination of active specific immunotherapy and chemotherapy as an adjuvant therapy in stage III colon cancer patients to assess the influence of 5-Fluorouracil/Leucovorin on anti-tumour immunity induced by autologous tumour cell vaccinations. Anti-tumour immunity was measured before and after chemotherapy by means of delayed type hypersensitivity reactions, taken 48 h after the third and the fourth vaccination. We also investigated the toxicity of this combined immuno-chemotherapy treatment. Delayed type hypersensitivity reactions before chemotherapy had a median size of 20.3 mm, while after chemotherapy delayed type hypersensitivity size was 18.4 mm (*P*=0.01), indicating that chemotherapy hardly affected anti-tumour immunity. The severity of ulcers at the BCG vaccination sites was comparable to previous studies. In 30% of the patients grade III or grade IV chemotherapy related toxicity was seen; this is comparable to what is normally observed after adjuvant chemotherapy alone. This study shows that the active specific immunotherapy-induced anti-tumour immune response is only minimally impaired by consecutive chemotherapy and that the combined treatment of stage III colon cancer patients with active specific immunotherapy and 5-Fluorouracil/Leucovorin does not cause unexpected toxicity.

*British Journal of Cancer* (2002) **86**, 1230–1234. DOI: 10.1038/sj/bjc/6600254 www.bjcancer.com

© 2002 Cancer Research UK

## 

Colon cancer is one of the most prevalent malignancies in the industrialised world, with approximately 240 000 new cases diagnosed each year in the USA and Western Europe. When colon cancer is detected early, in stages I or II (Dukes A or B), the 5 year survival rate with surgery alone is 60–90%, depending on the depth of invasion of the tumour into the bowel wall. When the cancer has spread to the regional lymph nodes, or stage III (Dukes C), the 5 year survival rate is 25–60%. Important prognostic factors in stage III colon carcinoma include the number of positive nodes, extra-nodal growth, invasion of adjacent organs, grade of histologic differentiation and post-operative CEA level. When distant metastases are diagnosed, stage IV (Dukes D), the 5 year survival rate is less than 5% (Cohen *et al*, 1997).

Currently most patients with stage III colon carcinoma receive an adjuvant treatment with 5-Fluorouracil (5-FU) and Leucovorin. Several large studies have shown that this chemotherapeutic regimen reduces the relative death rate of stage III patients by 30–40% (Wolmark *et al*, 1993; Francini *et al*, 1994; Moertel *et al*, 1995; IMPACT investigators, 1995; O'Connell *et al*, 1997).

Our efforts have focused on active specific immunotherapy (ASI), that makes use of the patient's own tumour to elicit a long-term anti-tumour immune response, as adjuvant therapy. Recently, we demonstrated in a randomised phase III study that adjuvant treatment with ASI, using autologous tumour cells and BCG, resulted in a significant reduction in the rate of recurrence in stages II and III colon cancer patients (Vermorken *et al*, 1999). Results were most pronounced in stage II patients, whereas in stage III colon cancer patients no clinically significant benefit was observed. The absence of a clinical effect in stage III colon cancer might be explained by the lack of statistical power of this study, as only 83 stage III patients were included. Furthermore, the residual tumour load in stage III patients is probably larger than in stage II patients, which could be relevant since it is known that ASI is more effective in a minimal residual disease setting (Hanna *et al*, 1979; Tamura *et al*, 1997; Baars *et al*, 2000).

In pre-clinical models ASI and chemotherapy were shown to have a synergistic anti-tumour effect. Apart from the capacity to directly destroy micro-metastases, ASI has been demonstrated to disrupt the characteristically compact structure of metastatic foci, enabling chemotherapy to reach deeper into the cancer tissue. In addition, chemotherapeutically killed tumour cells will disperse large amounts of intracellular antigens to an awaiting immune system. Furthermore, chemotherapy reduces the tumour burden, thereby increasing the chances of ASI subsequently eliminating the residual malignant cells (Hanna *et al*, 1982).

Several studies have demonstrated that the size of Delayed Type Hypersensitivity reactions (DTH) after autologous tumour cell vaccinations correlates strongly with recurrence and survival of cancer. The presence of a DTH response to tumour cells, 48 h after the vaccination regimen, is a measure of immunogenicity and reflects the adequacy of the vaccination and the general immune status of the patient. So far, DTH has been the only suitable parameter for the evaluation of anti-tumour immunity and the best early predictor of clinical efficacy of vaccination therapies (McCune *et al*, 1990; Harris *et al*, 2000; Baars *et al*, 2000; Clay *et al*, 2001).

Different strategies to test anti-tumour immunity have been investigated by other groups, often with disappointing results (Clay *et al*, 2001). Theoretically, peptide/MHC-tetramer staining of lymphocytes, with the purpose of detecting antigen specific cytotoxic T-lymphocytes, is the optimal method to evaluate the development of anti-cancer immunity (Altman *et al*, 1996). However, only a few tumour antigens have been identified for colon carcinoma, and it is still elusive which cancer antigens are most important for the development of a clinically relevant immune reaction. Moreover, studies investigating the correlation between the development of tetramer-positive CTL's and clinical prognosis have shown contradictory results (Lee *et al*, 1999; Fong *et al*, 2001; Haanen *et al*, 2001). Therefore, at this moment we consider the DTH response to be the most reliable parameter for the evaluation of the development of an immunological anti-tumour response in colon cancer patients after active specific immunotherapy with autologous tumour cells.

In preparation for a large randomised trial we investigated in this study whether the combined anti-cancer treatment with chemotherapy and ASI can be safely administered in colon cancer patients and whether 5-FU/Leucovorin affects the efficacy of ASI. Endpoints were toxicity and the size of DTH to autologous tumour cell vaccinations before and after chemotherapy.

## PATIENTS AND METHODS

### Patients

Patients undergoing a colon resection for a histologically proven or clinically suspected adenocarcinoma of the large bowel were selected. Distant metastases were excluded by means of pre-operative chest X-rays and liver ultrasound or CT-scanning. Patients with stage III colon cancer were eligible for this clinical trial. A WHO performance status of 0 or 1 was required. Patients with rectal cancer, perforated tumours, Crohn's disease, Ulcerative Colitis, previous malignancies, using immune suppressive medication, as well as pregnant and lactating women were excluded. Pathology reports were routinely reviewed on all patients. All patients gave written informed consent.

### Vaccine preparation

Six Dutch hospitals participated in this study. Surgery was performed in the referring hospital. Immediately after surgery part of the tumour tissue not required for pathological examination was excised and transferred to the vaccine production laboratory of the Vrije Universiteit Medical Center, Amsterdam. The tumour was dissociated as previously described (Hanna *et al*, 1979; Peters *et al*, 1979). After this procedure viability was tested using trypan blue. Cells were aliquotted (15–20×10^6^ viable cells per vial) and cryopreserved using a linear freezer. Vials were stored in liquid nitrogen until vaccination. For each patient four vaccines were produced.

### Vaccination procedure

**Figure 1:**
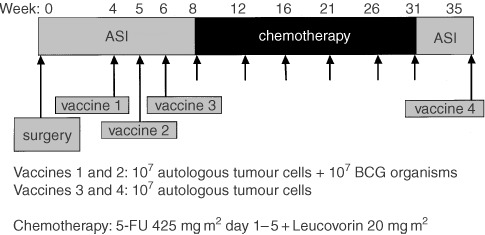
The treatment schedule that was used in this trial, showing that chemotherapy was preceded by 3 weekly tumour cell vaccinations. One month after the last gift of 5-FU a booster vaccination was administered.

Vaccination started 4–5 weeks after surgery to minimise the possible immune suppressive influence of surgery and anaesthesia. On the day of vaccination, after thawing, cells were counted and viability was assessed. For every vaccine 10^7^ viable autologous tumour cells were irradiated (200 Gy) and to the first two vaccines 10^7^ viable fresh-frozen BCG organisms were added. Vaccines were administered intra-cutaneously near sites of regional lymph nodes. The first two vaccines, containing BCG, were injected on the right and left anterior thigh respectively, at an interval of 1 week (Figure 1

). In the following week the third vaccine was given in the right upper arm. Eight months after the third vaccination, and at least 4 weeks after the last cycle of chemotherapy, a booster vaccination was administered in the left upper arm.

### Chemotherapy

Two weeks after the third vaccination, i.e. about 9 weeks after surgery, the first cycle of chemotherapy was given. Chemotherapy was administered in the referring hospital and normally consisted of 5-FU 425 mg m^2^ and Leucovorin 20 mg m^2^ as a bolus injection for 5 consecutive days, every 4 weeks for six cycles. In one of the participating hospitals, treating 12 of the patients described here, the chemotherapy was given in a 1 day per week schedule for 6 months, using 5-FU 600 mg m^2^ and Leucovorin 20 mg m^2^.

Patient charts were audited for all patients. Toxicity of the chemotherapy was measured using the National Cancer Institute Common Toxicity Criteria for Cancer Clinical Trials, version 2.0.

### DTH assessment

Response to the vaccines not containing BCG was measured by the size of induration 48 h after injection. Induration was assessed by the pen method (Sokal, 1975). For measurement of induration a line was drawn with a pen from 1 to 2 cm away from the margin of the skin test reaction towards the lesion. The pen is held at a 45° angle and moderate pressure is applied. The pen is advanced until resistance is met, indicating the edge of the area of induration. This procedure is performed in four directions. In this study the average of the two perpendicular diameters was used as the size of induration.

### Statistical analysis

Wilcoxon's matched pairs test was used for analysing differences between DTH-values. Calculations were made with SPSS 9.0 software.

## RESULTS

### Patients

Between March 1996 and December 1999 colon tumours of 104 stage III patients were processed. For 78 (75%) patients adequate vaccines could be produced. Fifty-six patients received the autologous vaccines of which 53 had finished their immunisation procedure at follow-up in June 2000. Reasons for not receiving vaccination therapy although having a stage III tumour were refusal of the patient to participate in this clinical trial (*n*=11), postoperative complications (*n*=4), the use of corticosteroid medication (*n*=2), alcoholic liver insufficiency (*n*=1), ulcerative colitis (*n*=1) and advanced age (*n*=3).

The median follow-up time was 21.7 months. Patient characteristics of all vaccinated stage III patients are shown in Table 1

**Table 1:**
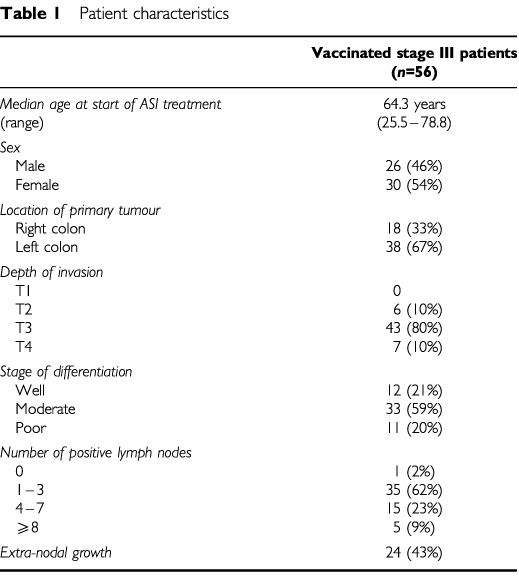
Patient characteristics

.

### Vaccine quality

The median weight of tumour received by our laboratory was 6.45 g (range 1.61–19.47 g). The median yield of tumour cells per gram of tissue was 17.9×10^6^ (range 4.0–39.7×10^6^).

### Side effects

In all patients ulcers developed at the site of the first and second vaccination, normally after a period of 2–3 weeks. Size of these ulcers ranged from 1 to 3 cm in diameter. All ulcers healed spontaneously after a median time of 2.6 months (range 0.8–7.1 months). Twenty-two patients (39%) noticed a fever and flu-like symptoms within 48 h after vaccination and eight patients (14%) mentioned tender regional lymph nodes during several days following the vaccinations. In Table 2

**Table 2:**
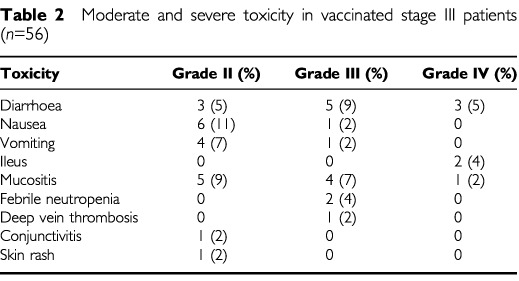
Moderate and severe toxicity in vaccinated stage III patients (*n*=56)

the toxicity occurring during chemotherapy treatment is presented. Seventeen patients (30%) experienced grade III or grade IV toxicity. A dose reduction was required in 20 patients (36%), of which eight (14%) could not finish all scheduled cycles due to toxicity.

One patient, a 60-year old male, developed grade IV mucositis and a paralytic ileus after the first cycle of chemotherapy. Colonoscopy showed diffuse ischaemia and ulcers, while the surgical anastomosis was intact. Shortly thereafter, a multiple organ failure syndrome developed and the patient died. Seven blood cultures showed no bacterial growth. Autopsy was not performed. Death of this patient is considered to result from toxicity of 5-FU.

### Delayed type hypersensitivity reaction (DTH)

**Figure 2:**
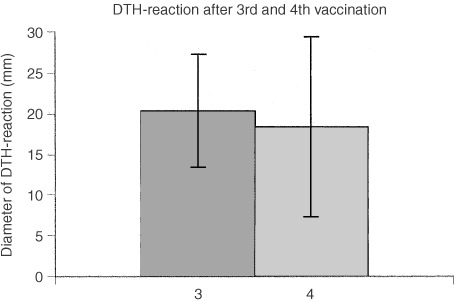
The diameter of the Delayed Type Hypersensitivity Reactions after the third and the fourth autologous tumour cell vaccination, measured 48 h after injection.

In each patient some form of local reaction was seen, 48 h after the third and fourth vaccinations. This consisted of erythema and induration. As shown in Figure 2

, the mean diameter of the induration after the third vaccination was 20.3 mm (s.d. 6.4). After the fourth vaccination this was 18.4 mm (s.d. 10.9). These values showed a statistically significant difference (*P*=0.01). In one patient ulceration occurred after the third vaccination, healing spontaneously within 3 weeks. The same was observed in another patient after the fourth vaccination.

## DISCUSSION

This is the first study using the combined treatment of chemotherapy and autologous tumour cell vaccinations for the adjuvant treatment of colon cancer. The most important result presented here is that the DTH-reaction after the fourth autologous tumour cell vaccination is still strong, despite the fact that 5-FU/Leucovorin was administered between the third and the fourth vaccination. This is important because DTH-reactions indicate the development of specific cellular immunity and correlate strongly with survival (McCune *et al*, 1990; Baars *et al*, 2000; Harris *et al*, 2000). A recently published study with autologous colon tumour vaccines has shown that DTH-reactions with a diameter exceeding 10 mm should be considered to reflect successful vaccination therapy and predict relatively good survival rates (Harris *et al*, 2000).

Thus, the chemotherapy regimen used in our protocol seems not to have a clinically important suppressive influence on the anti-tumour immunity induced by vaccination with tumour cells. However, other types of chemotherapy might have a more pronounced negative effect on anti-tumor immunity than 5-FU, so future combinations of immune therapy and chemotherapy will have to be carefully tested too, before being applied on large scale in a clinical setting.

Toxicity after the tumour cell vaccinations, consisting of ulcers after the first two injections and low grade fever in a minority of patients, was comparable to what we observed in our previous study, when no chemotherapy was used (Vermorken *et al*, 1999).

Side effects of the chemotherapy regimen were considerable, with grade III or grade IV toxicity occurring in 30% of patients. Gastrointestinal toxicity and mucositis were most prominent. One patient died shortly after receiving the first course of chemotherapy. Clinically, the most likely cause of death in this patient was acute mesenteric ischaemia, but this was not proven since an autopsy was not performed. Vascular toxicity is a known side-effect of 5-FU/Leucovorin, most often presenting like coronary vasospasms, but solitary bowel ischaemia has also been described (Fata *et al*, 1999). The precise mechanisms behind these circulatory changes are unknown (Robben *et al*, 1993). Overall, in this study the toxicity seen after chemotherapy was not different from the toxicity of previous studies using 5-FU/Leucovorin, and thus appears not to be influenced by the immunotherapy given in our clinical trial.

In conclusion, this study shows that the ASI-induced anti-tumour immune response is only minimally impaired by consecutive 5-FU/Leucovorin and that the combined treatment of stage III colon cancer patients with ASI and chemotherapy does not cause unexpected toxicity. Therefore, we recently started a randomised international phase III study investigating the efficacy of ASI combined with 5-FU+Leucovorin as an adjuvant treatment for stage III colon cancer patients.
